# Anatomical distribution of disease in pN1 prostate cancer with BCR post‐RP: A PSMA‐PET/CT‐based analysis

**DOI:** 10.1002/bco2.70123

**Published:** 2025-12-10

**Authors:** Lotte G. Zuur, Katelijne C. C. de Bie, Lieke Wever, Anne‐Claire Berrens, Jean‐Paul A. van Basten, Harm H. E. van Melick, Maarten L. Donswijk, Daniela E. Oprea‐Lager, Wouter V. Vogel, Henk G. van der Poel, André N. Vis, Pim J. van Leeuwen

**Affiliations:** ^1^ Department of Surgical Oncology (Urology) Netherlands Cancer Institute Amsterdam the Netherlands; ^2^ Department of Urology Amsterdam University Medical Centre Amsterdam the Netherlands; ^3^ Department of Urology Canisius Wilhelmina Hospital Nijmegen the Netherlands; ^4^ Department of Urology St. Antonius Hospital, Utrecht Nieuwegein the Netherlands; ^5^ Department of Nuclear Medicine Netherlands Cancer Institute Amsterdam the Netherlands; ^6^ Department of Medical Imaging Radboud University Medical Center Nijmegen the Netherlands; ^7^ Department of Radiation Oncology Netherlands Cancer Institute Amsterdam the Netherlands

**Keywords:** node‐positive, prostate cancer, prostate‐specific membrane antigen (PSMA), recurrence

## Abstract

**Objectives:**

To explore the distribution of metastatic disease in pN1 patients with biochemical recurrence (BCR) assessed by Prostate‐Specific Membrane Antigen‐Positron‐Emission/Computed Tomography (PSMA‐PET/CT).

**Patients and Methods:**

This multicentre, retrospective cohort study included 130 pN1 PCa patients with BCR (PSA ≥ 0.2 ng/ml) post‐RP with ePLND (2015–2022). All were preoperatively staged as molecular imaging (mi)N0M0 and underwent restaging PSMA‐PET/CT at BCR. Clinical and imaging data were analysed using Mann–Whitney U, chi‐square tests and Cox regression.

**Results:**

Median time to BCR was 9 months (IQR 3–18). Biochemical persistence (BCP) (PSA ≥ 0.1 ng/ml at first follow‐up) occurred in 66/130 (51%). At restaging PSMA‐PET/CT, median PSA was 0.33 ng/ml (IQR 0.24–0.65). PSMA‐PET/CT identified metastases in 63/130 (48%), with 37/63 (59%) having lymph node metastases (LNMs) limited to the ePLND template – 16/37 (43%) on the ipsilateral side of the positive resected node. Additionally, 26/63 (41%) had disease beyond the nodal template (miM+). Univariate Cox regression identified BCP as a predictor for any PSMA‐PET/CT‐detected recurrence, while a higher ISUP grade group at RP predicted miM+ disease.

**Conclusion:**

PSMA‐PET/CT identified PSMA‐expressing recurrent disease in nearly half of pN1 PCa patients at early BCR, with about half confined to the pelvis and a quarter beyond the pelvis. These anatomical patterns indicate that PSMA‐PET/CT can inform risk‐adapted salvage strategies, including pelvic radiotherapy for nodal recurrences and systemic treatment when disease extends beyond the pelvis.

## Abstract 2

We studied 130 men with prostate cancer whose lymph nodes contained cancer at the time of surgery. After surgery, all these patients showed signs that the cancer might be returning, and scans found possible cancer in nearly half of them. Those with more aggressive tumours were more likely to have cancer spread beyond the area where it first appeared.

## 1 INTRODUCTION

While advances in molecular imaging, particularly Prostate‐Specific Membrane Antigen‐Positron‐Emission/Computed Tomography (PSMA‐PET/CT), have significantly improved diagnostic accuracy compared to conventional imaging for prostate cancer (PCa), limited ability to detect micro metastases (<3 mm) remains a significant challenge for accurate staging.^1^, ^2^, ^3^, ^4^ Extended pelvic lymph node dissection (ePLND) during radical prostatectomy (RP) offers superior histological staging and is often performed in patients with higher‐risk disease. Although previous guidelines suggested ePLND when the estimated risk of nodal invasion exceeded 5% (e.g., Briganti nomogram), current international guidelines do not explicitly recommend PLND solely for staging purposes.^5^, ^6^ Pathological lymph node metastases (pN1) are identified in 10% to 30% of patients undergoing ePLND,^7^, ^8^, ^9^, ^10^ yet the extent to which ePLND contributes to disease control or long‐term outcomes in pN1 PCa patients remains unclear.^11^ Patients with pN1 disease represent a heterogeneous group with varying prognoses, influenced by factors such as the extent of nodal involvement and primary tumour stage.^12^, ^13^, ^14^ This heterogeneity complicates postoperative treatment decisions, which are currently guided by institutional practices and clinician preferences due to limited high‐level evidence.^5^, ^6^, ^9^, ^12^, ^15^ Guidelines offer options ranging from observation to adjuvant radiotherapy, with or without androgen deprivation therapy (ADT), all based on studies before the PSMA era.^16^

PSMA‐PET/CT has rapidly become the standard imaging tool for restaging PCa, offering high sensitivity for detecting recurrence even at low PSA levels.^17^, ^18^ Reflecting this, current guidelines now recommend PSMA‐PT/CT as the preferred modality for re‐staging.^5^, ^6^ Compared with conventional imaging, it enables earlier and more precise identification of recurrence sites, providing valuable insights into disease distribution. While challenges remain, including the risk of false positives, particularly in bone,^19^, ^20^ its overall diagnostic performance makes it an important tool for guiding management decisions in patients with pN1 disease after RP.

Building on these strengths, this study takes an exploratory approach to characterise PSMA‐expressing disease in pN1 PCa patients at the time of BCR. Beyond mapping the anatomical distribution of recurrence, we also investigate patterns of disease presentation, to better inform future risk‐adapted management strategies.

## 2 METHODS

This retrospective study was conducted in accordance with Good Clinical Practice guidelines and was approved by the Institutional Review Board of the Netherlands Cancer Institute (NCI) (IRBd22‐327). The study protocol was not prospectively registered; however, all analyses were predefined and conducted according to ethical standards. Data‐sharing agreements were established with Amsterdam University Medical Center (AUMC), Canisius Wilhelmina Hospital (CWZ) and St. Antonius Hospital (SAZ), which granted permission to share data for this study.

### 2.1 Patients

Patients were eligible for inclusion if they underwent robot‐assisted RP with ePLND and were staged as pN1 between 2015 and 2022 at the NCI, AUMC, SAZ or CWZ. An extended PLND was performed in patients with an estimated risk of lymph node invasion >7% according to the Briganti 2012 nomogram, which calculates risk based on preoperative clinical and pathological variables, including PSA level, clinical tumour stage, biopsy Gleason score and percentage of positive cores.^21^ Additionally, all included patients experienced BCR during follow‐up, defined as prostate‐specific antigen (PSA) ≥ 0.2 ng/ml. Included patients underwent pre‐operative and re‐staging PSMA‐PET/CT, and were pre‐operatively staged as molecular imaging (mi)N0M0.

Patients were excluded if preoperative PSMA PET/CT showed no initial focal PSMA uptake in the prostate (non‐PSMA‐expressing tumour [miT0]), if metastases were found on preoperative PSMA PET/CT (miN1 and/or miM1), if patients received any other PCa treatment, if post‐operative PSA levels were unknown or if re‐staging PSMA‐PET/CT was not performed.

Collected data included: age at time of RP, initial PSA level at diagnosis, clinical tumour stage (cT), date of RP, pathological International Society of Urological Pathology (ISUP) grade group, pathological tumour stage (pT), number of lymph nodes removed, location and pathological details of the positive resected lymph nodes, pathological margin status, follow‐up PSA levels, presence of biochemical persistence (BCP) (defined as PSA ≥ 0.1 ng/ml at first follow‐up) and PSMA‐PET imaging results.

### 2.2 Surgical PLND‐template and histopathology

RP included an ePLND according to the anatomical template defined by EAU, removing lymph nodes between the genitofemoral nerve, obturator fossa and iliac arteries up to the ureter crossing.^6^ Surgeons could extend dissection at their discretion. Surgery was performed using the robot‐assisted approach by experienced staff urologists from the participating hospitals.

Surgeons documented specimen locations in pathology reports, though details varied between institutions. Most reports distinguished prostate tissue from lymph nodes in the left and right hemipelvis. For analysis, lymph node locations were categorised as unilateral (left/right), bilateral or periprostatic. The ePLND specimens were analysed by respective institutional uropathologists per guidelines, assessing lymph node count, size and metastases. All included patients were classified as pathologically node‐positive (pN1).

### 2.3 Imaging protocol and interpretation

Each institution employed its own imaging protocols and PET/CT scanners, resulting in differences in injection dose, timing post‐injection, tracer selection and scan parameters, according to the EANM guideline.^22^ The following PSMA tracers were used: [68Ga]Ga‐PSMA‐11, [18F]DCFPyL, [18F]PSMA‐1007 and [18F]JK‐PSMA‐7. The tracers were synthesised according to Good Manufacturing Practices.

Experienced nuclear medicine physicians interpreted all pre‐operative and re‐staging scans in a clinical setting, reporting on primary and secondary lesions, miT stage, local recurrence after RP (miTr), lymph node metastases (LNM) (miN1/M1a) and distant metastases (miM1b/c) following E‐PSMA guidelines.^23^ Lesions with PSMA tracer uptake in a typical site of prostate cancer, with or without definitive findings on CT (E‐PSMA scores 4–5), were considered positive. All patients and corresponding PSMA‐PET/CT imaging results were discussed in multidisciplinary team meetings, and subsequent management (e.g., adjuvant or salvage therapy) was based on these findings. As not all lesions were confirmed by biopsy or additional imaging, no independent ground truth could be established.

LGZ and KdB reviewed re‐staging scans and reports to classify recurrences using PROMISE criteria.^24^ LNMs were categorised as within or outside the ePLND template, with the common iliac bifurcation as the boundary (miN1 below, miM1a above). Template recurrences were classified as unilateral, bilateral or local tumour recurrence (Tr+). Patients with both miM1b/c and miN1 or miM1a were categorised as miM1b/c. Recurrences were correlated with ePLND findings to assess distribution relative to pathological nodes.

### 2.4 Adjuvant therapy

Adjuvant treatment decisions were determined at multidisciplinary meetings, based on PSMA‐PET/CT findings and institutional practice. In our institution, when adjuvant treatment was indicated, patients received PSMA‐PET/CT‐guided early salvage radiotherapy. Further details on adjuvant treatment are beyond the scope of this study.

### 2.5 Statistical analysis

Continuous variables were assessed for normality using histograms. Non‐normally distributed data were summarised as median (inter‐quartile range [IQR]) and normally distributed data as mean (standard deviation [SD]). Categorical variables were summarised using frequencies and proportions. Descriptive statistics were used for patient characteristics and PSMA PET/CT detection. Group comparisons used Mann–Whitney U tests for continuous and chi‐square tests for categorical variables. Univariate Cox regression analyses were performed at 6, 12 and 24 months to identify predictors of PSMA‐expressing disease within and beyond the ePLND template, with the aim of describing temporal patterns of recurrence and characterising when patients developed early biochemical recurrence or detectable lesions. Due to the limited number of events, multivariable modelling was not feasible; thus, the analyses should be regarded as exploratory and hypothesis‐generating. Clinically relevant factors associated with PCa progression were included: initial PSA, ISUP grade group, pT‐stage, number of positive lymph nodes at ePLND, positive surgical margin, extranodal extension and BCP.^12^ Analyses were performed using SPSS version 30 (IBM, Armonk, NY, USA).

## 3 RESULTS

### 3.1 Baseline characteristics

In total, 130 pN1 patients with BCR after RP with ePLND for primary PCa were included in this study. The median age at RP of the entire cohort was 67 years (interquartile range [IQR] 59–72) and the median initial serum PSA level at diagnosis was 13 ng/ml (IQR 8–24). Most patients had pT3b (56%) and pT3a (32%) tumours, and the mean number of resected lymph nodes was 19 (standard deviation [SD] 8). The median size of the positive lymph nodes was 2 mm (IQR 2–4). BCP was present in 66 (51%) of patients. The median follow‐up time from RP with ePLND to last follow‐up was 35 months (IQR 25–51) and the median time to BCR was 9 months (IQR 3–18). The median PSA at re‐staging PSMA‐PET/CT was 0.33 (IQR 0.24–0.65). At re‐staging PSMA‐PET/CT for BCR, 67/130 (52%) patients had non‐PSMA‐expressing disease and 63/130 (48%) patients had PSMA‐expressing disease. Baseline clinical and tumour characteristics are presented for the entire cohort in Table 1, and separately for patients with non‐PSMA‐expressing and PSMA‐expressing scans in supplementary Table S1. Patients with PSMA‐expressing lesions had a higher number of positive lymph nodes excised during ePLND (*p* = 0.019) (Table S1).

**TABLE 1:** Clinical patient and pathological tumour characteristics.

	Total (n = 130)
Age, years, median (IQR)	67 (59–72)
iPSA, median (IQR)	13 (8–24)
pT‐stage, n (%)	
pT2a,b,c	13 (10)
pT3a	41 (32)
pT3b	73 (56)
pT4	2 (2)
ISUP grade group at RP, n (%)	
2	14 (11)
3	48 (37)
4	16 (12)
5	52 (40)
Surgical margin status, n (%)	
Positive	84 (65)
Negative	46 (35)
Number of lymph nodes removed, mean (SD)	19 (8)
Number of positive lymph nodes, median (IQR)	1 (1–2)
Size of positive lymph nodes in mm, median (IQR)	2 (2–4)
Location positive lymph node pathology, n (%)	
Unilateral left	35 (27)
Unilateral right	44 (34)
Both sides	45 (36)
Pre‐prostatic fat	4 (3)
PSA at re‐staging PSMA‐PET/CT, median (IQR)	0.33 (0.24–0.65)
PSMA tracer used for re‐staging PSMA‐PET/CT, n (%)	
^18^F‐DCFPyl‐PSMA	29 (22)
^18^F‐JK‐PSMA‐7	16 (12)
^18^F‐PSMA‐1007	19 (15)
^68^Ga‐PSMA‐11	66 (51)
BCP, n (%)	66 (51)
Follow‐up time in months, median (IQR)	35 (25–51)
Time to BCR in months, median (IQR)	9 (3–18)

Abbreviations: SD = standard deviation, iPSA = initial prostate‐specific antigen level, IQR = interquartile range, cT‐stage = clinical tumour stage, pT‐stage = pathological tumour stage, n = number, ISUP = International Society of Urological Pathology, RP = radical prostatectomy, PSMA‐PET/CT = prostate‐specific membrane antigen positron emission computed tomography, BCP = biochemical persistence, BCR = biochemical recurrence.

### 3.2 Distribution of PSMA‐expressing disease

**FIGURE 1:**
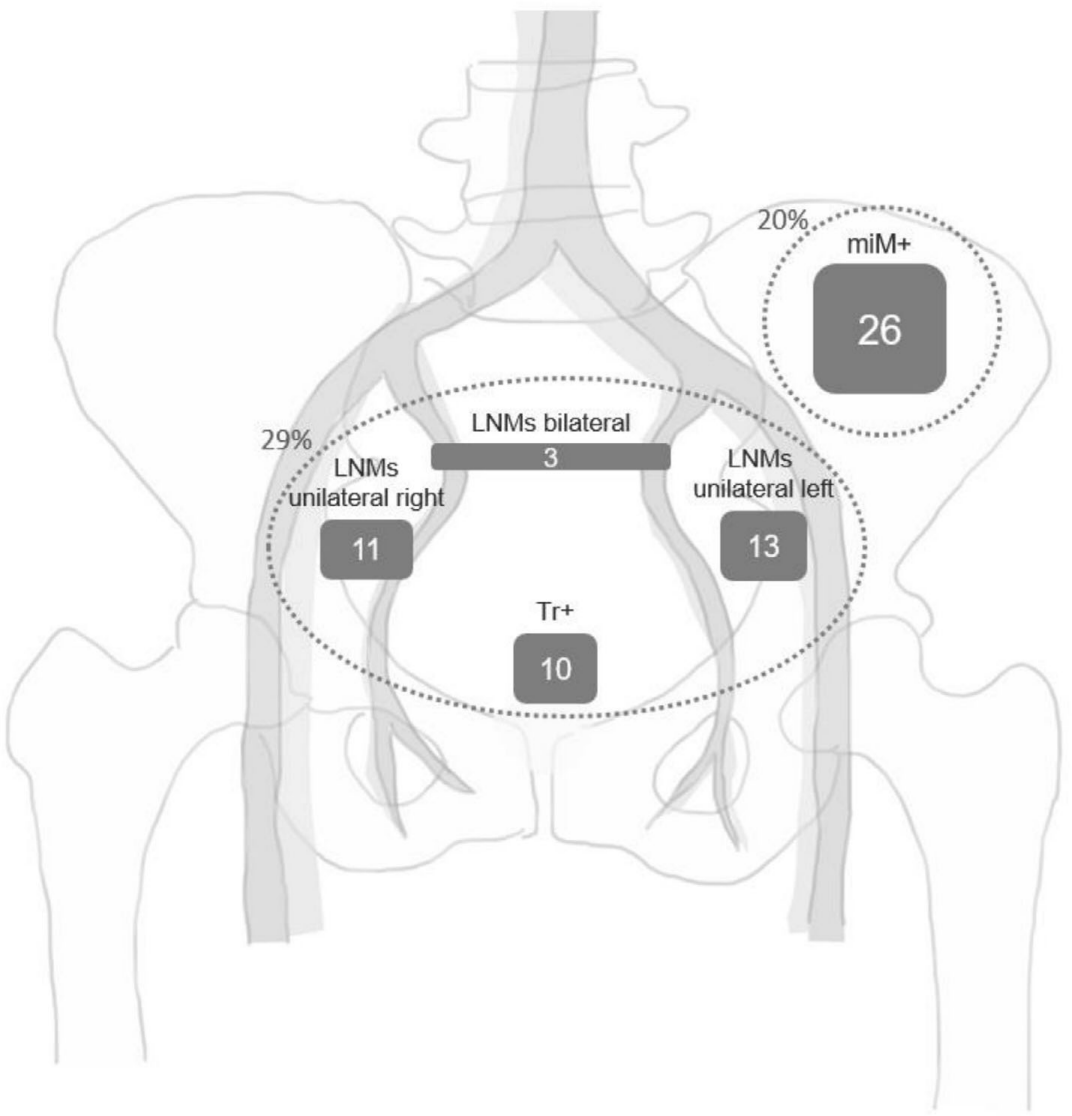
The distribution of PSMA‐avid lesions detected by PSMA‐PET/CT in pN1 PCa patients at early BCR. 37/130 (29%) patients had disease limited to the ePLND template and 26/130 (20%) outside of the template. The numbers within the squares indicate the number of patients. The Tr + includes all patients with Tr+, including those with both LNMs and Tr+. *LNMs = lymph node metastases, Tr+ = local recurrence of disease in the prostatic fossa and/or pre‐prostatic fat, miM+ = recurrent disease outside ePLND template PSMA‐PET/CT = prostate specific membrane antigen positron emission computed tomography, pN1 = pathological node‐positive, PCa = prostate cancer.*

PSMA‐PET/CT detected disease limited to the ePLND template in 37/130 (29%) patients and beyond the template in 26/130 (20%) patients (Table 2 and Figure 1). Within the ePLND template, miN1 disease was identified in 32/130 (24%), and miTr was observed in 10/130 (8%) patients. In 16/130 (12%) patients, the PSMA‐expressing lesion was ipsilaterally located with the resected positive lymph node. Among those with PSMA‐expressing disease outside the ePLND template, 5/130 (4%) patients had isolated miM1a disease, 16/130 (12%) had only miM1b/c disease and 5/130 (4%) patients had both miM1a and miM1b/c disease.

**TABLE 2:** Distribution of PSMA‐expressing disease.

	Total (n = 130)
LNMs limited to the ePLND template, n (%)	37 (29)
Location LNMs limited to the ePLND template, n (%)	
LNMs unilateral left	13 (10)
LNMs unilateral right	11 (9)
LNMs bilateral	3 (2)
miTr	8 (6)
miTr and LNM unilateral left	1 (1)
miTr and bilateral LNMs	1 (1)
Location of LNMs ipsilateral to the resected positive lymph node, n (%)	
Yes	16 (12)
No	6 (5)
Unknown	15 (12)
Recurrence outside ePLND template, n (%)	26 (20)
Isolated miM1a	5 (4)
Isolated miM1b/c	16 (12)
Both miM1a + miM1b/c	5 (4)
Recurrence both within and outside ePLND template, n (%)	13 (10)

Abbreviations: n = number, ePLND = extended pelvic lymph node dissection, LNMs = lymph node metastases, miTr = local recurrence in prostatic fossa, miM1a = extra‐pelvic lymph nodes, miM1b = bone metastasis, miM1c = non‐nodal visceral metastasis.

### 3.3 Subgroup analysis

Subgroup analyses were performed to identify statistically significant differences between non‐BCP and BCP patients (Table S2) and between patients with disease limited to the ePLND template versus those with disease outside the ePLND template (miM+) (Table S3). Among the 64 BCP patients, 34 had PSMA‐expressing disease, with 16 of them having disease outside the ePLND template on post‐operative PSMA‐PET/CT (Table S2). Compared to non‐BCP patients, BCP patients presented with higher initial PSA, higher PSA at re‐staging PSMA‐PET/CT and a shorter time to BCR (*p =* 0.016, *p =* <0.001 and *p =* <0.001 respectively) (Table S2). In addition, patients with disease outside the ePLND template had a higher median number of positive lymph nodes at ePLND than those with disease limited to the template (*p* = 0.037) (Table S3).

### 3.4 Cox regression analysis for predictors

Regression analysis at 6, 12 and 24 months, as well as during overall follow‐up, was performed to identify predictors of PSMA‐expressing disease (Table S4) and PSMA‐expressing disease outside the ePLND template (miM+) (Table S5). BCP was a statistically significant predictor of PSMA‐expressing disease at all follow‐up time points (Table S4) and of miM+ disease at 1‐year, 2‐year and overall follow‐up (Table S5). The ISUP grade group at RP was a significant predictor of PSMA‐expressing disease at the 2‐year follow‐up (p = 0.036), with grade groups 4–5 more frequently showing detectable or extra‐pelvic disease than grade groups 2–3, roughly corresponding to intermediate and high‐risk categories in the EAU risk group (Table S4). Grade group 4–5 was also associated with miM+ disease at both 2‐year and overall follow‐up (p = 0.029 and p = 0.029, respectively) (Table S5).

## 4 DISCUSSION

In this retrospective cohort of pN1 PCa patients with BCR, PSMA‐PET/CT identified two distinct groups: approximately half had disease confined to the pelvis, while a quarter had distant metastases. BCP was the strongest predictor of PSMA‐expressing recurrent disease, and higher ISUP grade at RP was associated with distant metastases at PSMA‐PET/CT in patients with early BCR. These findings suggest that PSMA‐PET/CT can help tailor salvage therapy by identifying patients suitable for local versus systemic treatment.

In our cohort, approximately half of PSMA‐expressing recurrences were located within the ePLND template, indicating that even extended dissections may leave residual pelvic disease. This finding confirms that lymph node removal alone may be insufficient for local tumour control. This finding aligns with the ongoing uncertainty regarding the oncological benefit of ePLND, as evidence supporting improved local control remains limited, and the EAU guideline panel did not incorporate ePLND into formal recommendations.^6^, ^25^, ^26^ Nevertheless, comprehensive dissection of pelvic nodes, including presacral, internal iliac and deep obturator regions, may have potential benefits that warrant further investigation. Further research is needed to clarify the role of ePLND in the era of PSMA‐PET/CT, whose findings should be interpreted with caution due to limitations such as potential false positives and undetected micro‐metastases. Interpretation of our results is further limited by the small sample size and the heterogeneous use of PSMA tracers, which precluded meaningful sub‐analysis by tracer type. A multicentre randomised trial is currently comparing RP with and without ePLND in patients at higher risk of lymph node involvement,^27^ and efforts should continue to refine the quality, completeness and effectiveness of lymph node dissection during RP.

Our results identified two distinct patient groups with different implications for treatment. About a quarter of patients had PSMA‐PET/CT–detectable distant metastases (miM+), for whom systemic therapy may be more appropriate than local radiotherapy, as local treatment alone is unlikely to achieve disease control. Conversely, nearly half of patients had no visible disease on PSMA‐PET/CT at re‐staging, raising uncertainty about the benefit of immediate adjuvant therapy versus the risk of overtreatment and toxicity.^28^ These findings suggest that PSMA‐PET/CT can help tailor early salvage strategies by identifying patients likely to benefit from systemic treatment and those for whom observation may be reasonable.

We found that PSMA‐PET/CT effectively differentiates between regional and distant metastatic disease at a median PSA level of 0.39 ng/ml, above the threshold typically considered for early salvage (radio)therapy (i.e., 0.25 ng/ml).^29^ We explored potential predictors for especially metastatic disease, but predictive patterns for regional or distant metastases remain unclear. BCP was the only predictor identified for both regional and distant metastases, without distinguishing between them. These analyses were univariate and exploratory, with no formal correction for multiple testing and multivariable analyses were not feasible due to limited event numbers. As such, results should be interpreted with caution and validated in independent cohorts. A tailored PSMA‐PET/CT‐guided approach could help identify patients whose disease remains limited to the pelvis and may benefit from local therapy, as well as those requiring systemic treatment in case of metastatic disease.

Our results are broadly similar to Huits et al., who reported PSMA‐expressing metastatic recurrences in two‐thirds of pN1 patients with BCR, with a quarter outside the pelvis.^30^ Interestingly, they found 84% of LNMs on PSMA‐PET/CT on the same side as the resected positive lymph node at ePLND, whereas we observed this in 16/37 (43%) of patients. Another study by Moschini et al. also analysed the recurrence patterns of pN1 PCa patients and found that a quarter of pN1 patients with BCR had PSMA‐positive recurrences on restaging PSMA‐PET/CT,^31^ about half the number observed in our study. The difference could be attributed to Moschini's cohort lacking PSMA‐PET/CT imaging and standardised ePLND procedures, which likely affected recurrence detection. Both studies have relatively small sample sizes, which may limit the precision of these estimates. Given the similar baseline characteristics of both cohorts, this discrepancy may stem from our multicentre setting, where variations in PSMA PET tracers and interobserver differences could play a role.

Strengths of our study include its focus on recurrence patterns specifically in pN1 patients and the use of PSMA‐PET/CT imaging, which is now considered the standard for detecting recurrent PCa.^6^ However, several limitations should be acknowledged. First, our study included multiple PSMA tracers and imaging systems across centres, with variable imaging protocols and potential interobserver differences. This heterogeneity may have influenced both detection and characterisation of recurrences, and represents a major limitation that affects the interpretability of pooled results. Second, the cohort of 130 pN1 patients is small, which limits statistical power and precision of the estimates. While achieving larger sample sizes in pN1 patients undergoing PSMA‐PET/CT is challenging, this small sample constrains our ability to perform detailed multivariable analyses and may limit generalisability. Moreover, due to the small sample size, meaningful sub‐analysis per tracer type was not feasible without compromising statistical power. Third, the study is retrospective in design, which limits causal inference. Fourth, differences in histopathological evaluation across institutions could have impacted lymph node assessments. Finally, the exploratory univariate analyses should be interpreted with caution due to the limited number of events.

Our findings support the potential of PSMA‐PET/CT to guide early salvage treatments, particularly to differentiate between pelvic‐confined recurrences and distant metastases, avoiding overtreatment. The absence of reliable predictors for recurrence location underscores the need for individualised treatment strategies and comprehensive patient monitoring. Future research should integrate PSMA‐PET/CT findings to enhance risk stratification and evaluate its impact on survival and quality of life. Collaboration across institutions to standardise imaging and pathological protocols will be important to enhance the reliability and generalisability of results.

## 5 CONCLUSION

In patients with pN1 PCa, restaging PSMA‐PET/CT at early BCR identified newly detected metastatic disease in nearly half of patients, whereof half were limited to the pelvis and a quarter showed distant metastases. These anatomical patterns indicate that PSMA‐PET/CT can inform risk‐adapted early salvage strategies, balancing the harms and benefits of early salvage radiotherapy.

## AUTHOR CONTRIBUTIONS

**Lotte G. Zuur:** Data acquisition; data analysis; writing—original draft preparation. **Katelijne C. C. de Bie:** Data acquisition; data analysis. **Lieke Wever:** Data acquisition. **Anne‐Claire Berrens:** Review. **Jean‐Paul A. van Basten:** Conceptualization; review. **Harm H. E. van Melick:** Conceptualization; review. **Maarten L. Donswijk:** Review. **Daniela E. Oprea‐Lager:** Review. **Wouter V. Vogel:** Review. **Henk G. van der Poel:** Review.**André N. Vis:** Conceptualization; supervision; review. **Pim J. van Leeuwen:** Conceptualization; supervision; review.

## CONFLICT OF INTEREST STATEMENT

The authors declare no conflict of interest.

## Supporting information


**Table S1.** Clinical patient and pathological tumour characteristics.
**Table S2.** Differences between non‐BCP and BCP patients.
**Table S3.** Differences between patients with recurrences limited to versus outside the ePLND template.
**Table S4.** Univariate Cox regression analysis of predictors for PSMA‐expressing disease after 6 months, 1 year, 2 years and overall follow‐up.
**Table S5.** Univariate Cox regression analysis of predictors for disease outside the ePLND template after 6 months, 1 year, 2 years and overall follow‐up.
