# Supplementary material for: Anatomical distribution of disease in pN1 prostate cancer with BCR post‐RP: A PSMA‐PET/CT‐based analysis
Source: BJUI Compass. 2025 Dec 10;6(12):e70123. doi: 10.1002/bco2.70123 (PMC12690614; doi:10.1002/bco2.70123)
Supplement: Supplementary file 1 — Table S1. Clinical patient and pathological tumour characteristics. Table S2. Differences between non‐BCP and BCP patients. Table S3. Differences between patients with recurrences limited to versus outside the ePLND template. Table S4. Univariate Cox regression analysis of predictors for PSMA‐expressing disease after 6 months, 1 year, 2 years and overall follow‐up. Table S5. Univariate Cox regression analysis of predictors for disease outside the ePLND template after 6 months, 1 year, 2 years and overall follow‐up. [file BCO2-6-e70123-s001.docx]

| *Supplementary Table S1. Clinical patient and pathological tumour characteristics* | | | | |
| --- | --- | --- | --- | --- |
|  | | Non PSMA-expressing  (n=67, 52%) | PSMA-expressing  (n=63, 48%) | *p* value |
| Age, years, median (IQR) | | 67 (59-71) | 66 (57-72) | 0.828 |
| iPSA, median (IQR) | | 16 (8-28) | 12 (8-23) | 0.407 |
| pT-stage, n (%) | |  |  | 0.642 |
|  | pT2a,b,c | 9 (13) | 4 (6) |  |
|  | pT3a | 24 (36) | 17 (27) |  |
|  | pT3b | 33 (49) | 40 (64) |  |
|  | pT4 | 1 (2) | 2 (3) |  |
| ISUP grade group at RP, n (%) | |  |  | 0.071 |
|  | 2 | 8 (12) | 6 (10) |  |
|  | 3 | 31 (46) | 17 (27) |  |
|  | 4 | 8 (12) | 8 (13) |  |
|  | 5 | 20 (30) | 32 (51) |  |
| Surgical margin status, n (%) | |  |  | 0.584 |
|  | Positive | 45 (67) | 39 (62) |  |
|  | Negative | 22 (33) | 24 (38) |  |
| Number of lymph nodes removed, mean (SD) | | 20 (9) | 18 (6) | 0.319 |
| Number of positive lymph nodes, median (IQR) | | 1 (1-2) | 2 (1-3) | **0.019** |
| Size of positive lymph nodes in mm, median (IQR) | | 2 (2-4) | 2 (2-5) | 0.358 |
| Location positive lymph node pathology, n (%) | |  |  | 0.240 |
|  | Unilateral left | 19 (28) | 16 (25) |  |
|  | Unilateral right | 27 (40) | 17 (27) |  |
|  | Both sides | 18 (27) | 27 (43) |  |
|  | Pre-prostatic fat | 2 (3) | 2 (3) |  |
| PSA at re-staging PSMA-PET/CT, median (IQR) | | 0.30 (0.24-0.45) | 0.39 (0.24-1.05) | 0.074 |
| BCP, n (%) | | 30 (45) | 34 (54) | 0.380 |
| Follow-up time in months, median (IQR) | | 34 (23-52) | 35 (25-50) | 0.995 |
| Time to BCR in months, median (IQR) | | 10 (3-18) | 8 (3-16) | 0.499 |
| Abbreviations: SD = standard deviation, iPSA = initial prostate specific antigen level, IQR = inter quartile range, cT-stage = clinical tumour stage, pT-stage = pathological tumour stage, n = number, ISUP = International Society of Urological Pathology, RP = radical prostatectomy, PSMA-PET/CT = prostate specific membrane antigen positron emission computed tomography, BCP = biochemical persistence, BCR = biochemical recurrence | | | | |

| *Supplementary Table S2*. *Differences between non-BCP and BCP patients* | | | | |
| --- | --- | --- | --- | --- |
| Variables | | Non-BCP  (n=66) | BCP (n=64) | *p-*value |
| iPSA, median (IQR) | | 11 (8-19) | 19 (9-35) | **0.016** |
| pT-stage, n (%) | |  |  | 0.817 |
| pT2a,b,c |  | 8 (12) | 5 (8) |  |
| pT3a |  | 19 (29) | 22 (34) |  |
| pT3b |  | 38 (58) | 36 (56) |  |
| pT4 |  | 1 (2) | 1 (2) |  |
| ISUP at RP, n (%) | |  |  | 0.055 |
| 2 |  | 9 (14) | 5 (8) |  |
| 3 |  | 30 (46) | 18 (28) |  |
| 4 |  | 5 (8) | 11 (17) |  |
| 5 |  | 22 (33) | 30 (47) |  |
| Number of positive lymph nodes at ePLND, median (IQR) | | 1 (1-2) | 2 (1-3) | 0.068 |
| Size of positive resected lymph node in mm, median (IQR) | | 2 (2-4) | 2 (2-4) | 0.303 |
| Positive surgical margin, n (%) | | 40 (61) | 44 (69) | 0.363 |
| Extranodal extension of pathological lymph node, n (%) | | 19 (29) | 21 (33) | 0.084 |
| PSA at re-staging PSMA-PET/CT, median (IQR) | | 0.27 (0.22-0.39) | 0.46 (0.30-1.78) | **<0.001** |
| Location of recurrence, n (%) | |  |  | 0.348 |
| Non PSMA-expressing |  | 37 (56) | 30 (47) |  |
| Limited to ePLND template |  | 19 (29) | 18 (28) |  |
| Outside ePLND template |  | 10 (15) | 16 (25) |  |
| Time to BCR in months, median (IQR) | | 16 (12-26) | 3 (2-5) | **<0.001** |
| Abbreviations: BCP = biochemical persistence, iPSA = initial prostate specific antigen level, ISUP = International Society of Urological Pathology, RP = radical prostatectomy, ePLND = extended pelvic lymph node dissection, BCP = biochemical persistence | | | | |

| *Supplementary Table S3. Differences between patients with recurrences limited to versus outside the ePLND template* | | | | |
| --- | --- | --- | --- | --- |
| Variables | | Limited to ePLND  (n=37) | miM+ (n=26) | *p-*value |
| iPSA, median (IQR) | | 11 (8-21) | 14 (6-33) | 0.396 |
| pT-stage, n (%) | |  |  | 0.105 |
| pT2a,b,c |  | 4 (11) | 0 (0) |  |
| pT3a |  | 11 (30) | 6 (23) |  |
| pT3b |  | 22 (60) | 18 (69) |  |
| pT4 |  | 0 (0) | 2 (7) |  |
| ISUP grade group at RP, n (%) | |  |  | 0.327 |
| 2 |  | 3 (8) | 3 (12) |  |
| 3 |  | 13 (35) | 4 (15) |  |
| 4 |  | 5 (14) | 3 (12) |  |
| 5 |  | 16 (43) | 16 (62) |  |
| Number of positive lymph nodes at ePLND, median (IQR) | | 1 (1-3) | 2 (1-3) | **0.037** |
| Positive surgical margin, n (%) | | 20 (54) | 19 (73) | 0.188 |
| Extranodal extension of pathological lymph node, n (%) | | 10 (27) | 10 (39) | 0.318 |
| BCP, n (%) | | 18 (49) | 16 (62) | 0.442 |
| Time to BCR in months, median (IQR) | | 12 (4-20) | 5 (2-16) | 0.213 |
| Abbreviations: ePLND = extended pelvic lymph node dissection, miM+ = recurrent disease outside the ePLND template, iPSA = initial prostate specific antigen level, ISUP = International Society of Urological Pathology, RP = radical prostatectomy, BCP = biochemical persistence | | | | |

| *Supplementary Table S4. Univariate Cox regression analysis of predictors for PSMA-expressing disease after 6 months, 1 year, 2 years, and overall follow-up* | | | | | | | | | |
| --- | --- | --- | --- | --- | --- | --- | --- | --- | --- |
| Variables | | At 6-months follow-up  (n=22) | | At 1 year follow-up  (n=36) | | At 2 year follow-up  (n=54) | | Overall  (n=63) | |
|  | | HR (95% CI) | *p* value | HR (95% CI) | *p* value | HR (95% CI) | *p* value | HR (95% CI) | *p* value |
| Age at RP | | 0.98 (0.94-1.03) | 0.474 | 0.99 (0.95-1.03) | 0.573 | 1.00 (0.97-1.04) | 0.848 | 1.00 (0.97-1.03) | 0.946 |
| iPSA | | 1.35 (0.81-2.25) | 0.224 | 1.30 (0.86-1.96) | 0.207 | 1.32 (0.94-1.84) | 0.106 | 1.31 (0.94-1.82) | 0.114 |
| ISUP at RP | |  |  |  |  |  |  |  |  |
| 2 and 3 |  | ref | ref | ref | ref | ref | ref | ref | ref |
| 4 and 5 |  | 0.95 (0.40-2.26) | 0.908 | 2.17 (0.99-4.78) | 0.054 | 1.88 (1.04-3.39) | **0.036** | 1.58 (0.93-2.98) | 0.092 |
| pT-stage | |  |  |  |  |  |  |  |  |
| pT2a, pT2b and pT2c |  | ref | ref | ref | ref | ref | ref | ref | ref |
| pT3a |  | 0.78 (0.09-7.00) | 0.826 | 1.01 (0.22-4.66) | 0.994 | 1.00 (0.29-3.50) | 0.995 | 0.92 (0.31-2.75) | 0.883 |
| pT3b and pT4 |  | 1.66 (0.22-12.46) | 0.624 | 1.41 (0.33-5.94) | 0.644 | 1.37 (0.42-4.45) | 0.601 | 1.07 (0.38-2.99) | 0.905 |
| Number of positive lymph nodes at ePLND | | 1.08 (0.92-1.29) | 0.351 | 1.11 (0.99-1.25) | 0.074 | 1.09 (0.96-1.23) | 0.172 | 0.07 (0.94-1.21) | 0.313 |
| Positive surgical margin | | 1.13 (0.47-2.69) | 0.784 | 1.04 (0.53-2.03) | 0.911 | 1.22 (0.70-2.14) | 0.484 | 1.16 (0.69-1.94) | 0.580 |
| Extranodal extension of pathological lymph node | | 1.00 (1.00-1.00) | 0.556 | 1.00 (1.00-1.00) | 0.107 | 1.00 (1.00-1.00) | 0.078 | 1.00 (1.00-1.00) | 0.078 |
| BCP | | 25.41 (3.41-189.39) | **0.002** | 13.31 (5.03-35.26) | **<0.001** | 4.97 (2.76-8.98) | **<0.001** | 4.29 (2.48-7.44) | **<0.001** |
| Abbreviations: iPSA = initial prostate specific antigen level, IQR = inter quartile range, cT-stage = clinical tumour stage, pT-stage = pathological tumour stage, n = number, ISUP = International Society of Urological Pathology, RP = radical prostatectomy, PSMA-PET/CT = prostate specific membrane antigen positron emission computed tomography, BCP = biochemical persistence, BCR = biochemical recurrence | | | | | | | | | |

| *Supplementary Table S5. Univariate Cox regression analysis of predictors for disease outside the ePLND template after 6 months, 1 year, 2 years, and overall follow-up* | | | | | | | | | |
| --- | --- | --- | --- | --- | --- | --- | --- | --- | --- |
| Variables | | At 6-months follow-up  (n=10) | | At 1 year follow-up  (n=17) | | At 2 year follow-up  (n=24) | | Overall  (n=26) | |
|  | | HR (95% CI) | *p* value | HR (95% CI) | *p* value | HR (95% CI) | *p* value | HR (95% CI) | *p* value |
| Age at RP | | 1.02 (0.95-1.10) | 0.616 | 1.03 (0.97-1.10) | 0.332 | 1.01 (0.96-1.07) | 0.767 | 1.01 (0.96-1.07) | 0.725 |
| iPSA | | 1.08 (0.56-2.07) | 0.829 | 1.11 (0.69-1.81) | 0.663 | 1.14 (0.76-1.72) | 0.516 | 1.12 (0.75-1.67) | 0.594 |
| ISUP at RP | |  |  |  |  |  |  |  |  |
| 2 and 3 |  | ref | ref | ref | ref | ref | ref | ref | ref |
| 4 and 5 |  | 1.58 (0.36-7.46) | 0.563 | 3.66 (0.83-16.15) | 0.086 | 3.45 (1.14-10.46) | **0.029** | 3.45 (1.14-10.46) | **0.029** |
| pT-stage | |  |  |  |  |  |  |  |  |
| pT3a |  | ref | ref | ref | ref | ref | ref | ref | ref |
| pT3b and pT4 |  | 3.44 (0.43-27.18) | 0.242 | 2.09 (0.60-7.31) | 0.249 | 1.26 (0.49-3.21) | 0.635 | 1.26 (0.49-3.21) | 0.635 |
| Number of positive lymph nodes at ePLND | | 1.15 (0.85-1.56) | 0.380 | 1.22 (0.97-1.53) | 0.087 | 1.17 (0.94-1.46) | 0.168 | 1.17 (0.94-1.46) | 0.168 |
| Positive surgical margin | | 0.58 (0.16-2.07) | 0.404 | 0.62 (0.23-1.68) | 0.347 | 0.51 (0.19-1.32) | 0.162 | 0.51 (0.19-1.32) | 0.162 |
| Extranodal extension of pathological lymph node | | 1.00 (1.00-1.00) | 0.512 | 1.00 (1.00-1.00) | 0.431 | 1.00 (1.00-1.00) | 0.247 | 1.00 (1.00-1.00) | 0.247 |
| BCP | | 7.76 (0.98-61.52) | 0.052 | 10.44 (2.32-47.04) | **0.002** | 4.74 (1.82-12.30) | **<0.001** | 4.74 (1.82-12.30) | **<0.001** |
| Abbreviations: iPSA = initial prostate specific antigen level, IQR = inter quartile range, cT-stage = clinical tumour stage, pT-stage = pathological tumour stage, n = number, ISUP = International Society of Urological Pathology, RP = radical prostatectomy, PSMA-PET/CT = prostate specific membrane antigen positron emission computed tomography, BCP = biochemical persistence, BCR = biochemical recurrence | | | | | | | | | |
